# Mesenchymal stromal (stem) cells suppress pro-inflammatory cytokine production but fail to improve survival in experimental staphylococcal toxic shock syndrome

**DOI:** 10.1186/1471-2172-15-1

**Published:** 2014-01-14

**Authors:** Hani Kim, Ilyse Darwish, Maria-Fernanda Monroy, Darwin J Prockop, W Conrad Liles, Kevin C Kain

**Affiliations:** 1 Sandra A. Rotman Laboratories, Sandra Rotman Centre for Global Health, University Health Network-Toronto General Hospital, University of Toronto, Toronto, M5G 1 L7, Canada; 2 Tropical Disease Unit, Division of Infectious Diseases, Department of Medicine, University of Toronto, Toronto, ON, Canada; 3 Faculty of Medicine, Institute of Medical Science, University of Toronto, Toronto, ON, Canada; 4 University Health Network, Animal Resource Centre, Toronto, ON, Canada; 5 Institute of Regenerative Medicine, Texas A&M Health Science Center, College of Medicine at Scott & White, Temple, TX, USA; 6 Department of Medicine, University of Washington, Seattle, WA, USA

## Abstract

**Background:**

Toxic shock syndrome (TSS) is caused by an overwhelming host-mediated response to bacterial superantigens produced mainly by *Staphylococcus aureus* and *Streptococcus pyogenes*. TSS is characterized by aberrant activation of T cells and excessive release of pro-inflammatory cytokines ultimately resulting in capillary leak, septic shock, multiple organ dysfunction and high mortality rates. No therapeutic or vaccine has been approved by the U.S. Food and Drug Administration for TSS, and novel therapeutic strategies to improve clinical outcome are needed. Mesenchymal stromal (stem) cells (MSCs) are stromal cells capable of self-renewal and differentiation. Moreover, MSCs have immunomodulatory properties, including profound effects on activities of T cells and macrophages in specific contexts. Based on the critical role of host-derived immune mediators in TSS, we hypothesized that MSCs could modulate the host-derived proinflammatory response triggered by *Staphylococcal enterotoxin B* (SEB) and improve survival in experimental TSS.

**Methods:**

Effects of MSCs on proinflammatory cytokines in peripheral blood were measured in wild-type C57BL/6 mice injected with 50 μg of SEB. Effects of MSCs on survival were monitored in fatal experimental TSS induced by consecutive doses of D-galactosamine (10 mg) and SEB (10 μg) in HLA-DR4 transgenic mice.

**Results:**

Despite significantly decreasing serum levels of IL-2, IL-6 and TNF induced by SEB in wild-type mice*,* human MSCs failed to improve survival in experimental TSS in HLA-DR4 transgenic mice. Similarly, a previously described downstream mediator of human MSCs, TNF-stimulated gene 6 (TSG-6), did not significantly improve survival in experimental TSS. Furthermore, murine MSCs, whether unstimulated or pre-treated with IFNγ, failed to improve survival in experimental TSS.

**Conclusions:**

Our results suggest that the immunomodulatory effects of MSCs are insufficient to rescue mice from experimental TSS, and that mediators other than IL-2, IL-6 and TNF are likely to play critical mechanistic roles in the pathogenesis of experimental TSS.

## Background

Toxic shock syndrome (TSS) is a potentially fatal disease characterized by systemic capillary leak, commonly associated with hypoalbuminaemia, edema, hypotension, acute respiratory distress syndrome, and multiple organ dysfunction syndrome [1]. TSS is induced by exposure to bacterial superantigens produced predominantly by Gram-positive cocci, especially *Staphylococcus aureus* and *Streptococcus pyogenes*[1,2]*.* Conventional antigens are processed by antigen presenting cells (APC) into small peptides and presented within the MHC class II molecule on the surface of APCs to T cells. As a result, only a small fraction (<0.01%) of host T cell clones become activated [1]. In contrast, superantigens bypass antigen processing and bind directly to MHC class II/T cell receptor as whole antigens, activating up to 25% of total T cells in the host [1,3]. This results in excessive and uncoordinated production and release of pro-inflammatory cytokines, such as TNF, IL-6, IFNγ, IL-2, and ΙL-1β, which have been implicated in the pathogenesis of TSS [4-7], including capillary leak, septic shock, multiple organ dysfunction and death.

While several experimental therapeutics are being investigated, none has been approved by the U.S. Food and Drug Administration (FDA) for the treatment of TSS. As a result, mortality remains high especially in streptococcal TSS (30-50% mortality) compared to staphylococcal TSS (5-10% mortality) [8-10]. In addition, multiple potential routes of exposure, including epithelial surfaces, intestinal mucosa and inhalation, make superantigens a candidate for use in biological warfare [11]. Current clinical management for TSS mainly involves supportive therapy incorporating fluid resuscitation and vasopressors, and appropriate antibiotics [1]. Overall, there is an urgent need for a therapeutic strategy that targets the pathological process of TSS.

Mesenchymal stem cells (MSC) are a heterogenous subset of non-hematopoietic pluripotent stromal cells that can differentiate into multiple cell types of mesenchymal lineage (i.e., osteoblasts, chondroblasts and adipocytes) [12]. MSCs have been reported to improve tissue injury arising from multiple causes, including sepsis, acute renal failure, acute myocardial infarction and acute lung injury [13-16]. While the beneficial effects of MSCs were initially attributed to their pluripotency, the contribution of MSCs to tissue repair through engraftment and transdifferentiation into functionally relevant tissues remains unclear [17]. Increasing evidence indicates that MSCs can exert profound immunomodulatory effects that contribute mechanistically to the attenuation of tissue injury via suppression of immune effector cells, including T cells and macrophages, resulting in decreased production of proinflammatory cytokines and chemokines [18-22].

Based on the critical role played by the host immune response in the pathogenesis of TSS, we hypothesized that MSCs would decrease inflammation and improve survival in experimental TSS. Human MSCs significantly reduced the serum levels of IL-2, IL-6 and TNF, triggered by SEB in wild-type mice, while IFNγ was unaffected by hMSCs. Importantly, MSCs either human or mouse failed to improve survival in experimental TSS suggesting that their immunosuppressive effects are insufficient to reduce mortality in this model.

## Methods

### Reagents

SEB and D-(+)-galactosamine hydrochlorde (D-gal) were obtained from Sigma Aldrich (Oakville, ON, Canada). Recombinant human TSG-6 (rhTSG-6) was purchased from R&D Systems (Minneapolis, MN, USA). rhTSG-6 (30 μg/mouse) was administered to mice one hour prior to the D-gal injection.

### Mice

Wild-type male 8–10 week old C57BL/6 mice (Jackson Laboratory, Bar Harbor, Maine, USA) were used for the measurement of cytokines after SEB injection. For all survival analyses, 8–10 week old male C57BL/6 mice transgenic for HLA-DR4 (Taconic Farms, Inc., New York, NY, USA) were used [23].

TSS was induced in mice transgenic for HLA-DR4 by intraperitoneal injection of 10 mg of D-(+)-galactosamine hydrochlorde (Sigma-Aldrich Canada Ltd., Oakville, ON, Canada) followed, 1 hour later, by intraperitoneal injection of SEB (10 μg/mouse, Sigma-Aldrich Canada Ltd., Oakville, ON, Canada) [24]. Mice were monitored every 30 minutes during the first 5 hours, and continually once lethargy became apparent and progressive, at which time the mice were euthanized.

### Ethics statement

All experimental procedures were performed in accordance with the Canadian Council on Animal Care Guidelines, and were approved by the Toronto General Hospital Animal Care Committee at the University Health Network, Toronto, Canada.

### MSC culture

Frozen vials of murine MSCs (mMSCs) and human MSCs (hMSCs) were obtained from the Texas A&M Health Science Center College of Medicine Institute for Regenerative Medicine at Scott & White (Temple, TX, USA), under the auspices of a National Institutes of Health/National Centre for Research Resources (NIH/NCRR) grant (#P40RR017447). All MSCs were reported by the Center as meeting MSC- defining criteria proposed by the International Society for Cellular Therapy (ISCT) [25]. mMSCs (isolated from male C57BL/6 mice) were thawed and plated for 24 hours in Complete Growth Media (i.e., α-MEM without ribonucleosides or deoxyribonucleosides and supplemented with antibiotics, 10% fetal bovine serum (FBS) (Atlanta Biologicals, Miami, FL, USA) and 10% horse serum (Gibco, Carlsbad, CA, USA)). hMSCs (isolated from a 24 year old male donor) were thawed and plated for 24 hours in α-MEM, without ribonucleosides or deoxyribonucleosides, supplemented with 2mM L-glutamine, penicillin and streptomycin and 16.5% FBS. After 24 hours, mMSCs or hMSCs were trypsinized and re-plated at 60 cells/cm^2^. mMSCs/hMSCs were incubated for each subsequent passage until they reached 70% confluency. Passage 6 (P6) mMSCs or Passage 3 (P3) hMSCs were washed and resuspended in PBS. 2.5 × 10^5^ hMSCs or mMSCs or PBS alone was administered into mice intravenously via tail vein one hour prior to induction of TSS (hMSCs, mMSCs) or three hours after induction of TSS (mMSCs). For ex-vivo IFNγ pre-treatment of mMSCs, mMSCs were incubated in complete growth media containing 100 U/ml of mouse recombinant IFNγ for 3 hours, washed and resuspended in PBS before being injected into mice one hour before induction of TSS.

### Differentiation assays

MSCs were differentiated into adipocytes, osteocytes and chondrocytes using the Mouse MSC Functional Identification kit for murine MSCs (R&D Systems, Minneapolis, MN, USA) and StemPro Differentiation Kit (Gibco, Carlsbad, CA, USA) for human MSCs according to the manufacturers’ protocols. Briefly, MSCs were cultured in 12-well plates in α-MEM containing 20% FBS (Atlanta Biologicals, Miami, FL, USA), L-glutamine, penicillin and streptomycin until they reached 100% confluency for adipocytic differentiation, and 50-70% confluency for osteocytic and chondrocytic differentiation. MSCs were cultured in adipogenic, osteogenic or chondrogenic media for 7 – 21 days before being prepared for lineage-specific stains. Adipocytic differentiation was confirmed by staining with Oil Red O (Sigma-Aldrich Canada Ltd., Oakville, ON, Canada) as previously described [26]. Briefly, cells were washed with PBS and fixed in 10% formalin for 45 minutes. Fixed cells were incubated in 60% isopropanol for 5 minutes before being incubated in a freshly prepared Oil Red O solution for 15 minutes. Differentiation to osteocytes was confirmed by Alizarin Red S staining (Sigma-Aldrich Canada Ltd., Oakville, ON, Canada) [26]. Cells were washed and fixed as described above. Fixed cells were stained with 2% Alizarin Red S solution (w/v, pH4.2) for 20 minutes. For chondrocytic differentiation, spheroids of 3×10^5^ MSCs were allowed to form overnight in α-MEM media containing FBS, L-glutamine and antibiotics in 15mL conical polypropylene tubes. After being incubated in chondrogenic differentiation media for 21 days, each spheroid in 200 μl media was transferred to a cytospin sample chamber (Thermo Scientific, Mississauga, ON, Canada) attached to a glass slide, and centrifuged at 800 RPM for 10 minutes. The cells on glass slides were washed with PBS and fixed in 10% formalin for 1 hour before being incubated with 0.03% (w/v) Alcian Blue 8 GX (Sigma-Aldrich Canada Ltd., Oakville, ON, Canada) prepared in 60% ethanol and 40% acetic acid. After being stained overnight, cells were washed in destaining solution containing 60% ethanol and 40% acetic acid. Images of the stained cytospins were obtained by using a digital camera. Microscopic images of the stained chondrocytes were obtained by using a phase contrast microscope (oil immersion, magnification ×100).

### Serum cytokine measurement

Wild-type C57BL/6 mice were injected with 50 μg of SEB, and serum was collected 2, 4, 6, 8, 10, 12 and 24 hours later via cardiac puncture. Serum levels of IL-2, IL-6, TNF and IFNγ were measured by specific ELISAs (for IL-2 and IL-6, R&D Systems, Minneapolis, MN, USA; for TNF and IFNγ, eBioscience, San Diego, CA, USA).

### Statistical analysis

Statistical analyses were performed using the GraphPad Prism software (LaJolla, CA, USA). Statistical significance for survival studies was assessed by the log-rank test. Comparison of two groups at multiple time-points was performed by 2-Way ANOVA, and for a single time-point, by Mann–Whitney. In all cases, a p-value < 0.05 was considered significant.

## Results

### Administration of hMSCs suppresses proinflammatory cytokine production induced by SEB *in vivo*

**Figure 1:**
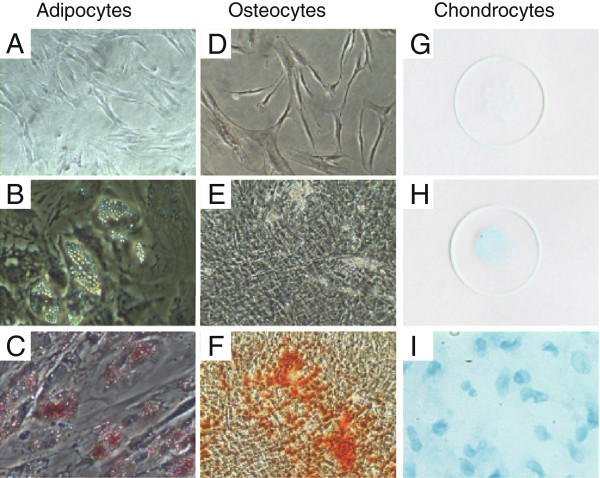
**Differentiation of Passage 3 hMSCs into adipocytes, osteocytes and chondrocytes.** Phase-contrast microscopy images of: adipogenic differentiation (**A**-**C**; Oil Red O staining of undifferentiated mMSCs **(A)** and differentiated adipocytes **(C)**; unstained differentiated adipocytes **(B)**); osteogenic differentiation (**D**-**F**; Alizarin Red S staining of undifferentiated mMSCs **(D)** and differentiated osteocytes **(F)**; unstained differentiated osteocytes **(E)**). **(G-I)** Alcian Blue staining of cytospins prepared from undifferentiated mMSCs **(G)** and differentiated chondrocytes **(H)**; phase-contrast microscopy image of the Alcian Blue stained cytopin of differentiated chondrocytes **(I)**.

**Figure 2:**
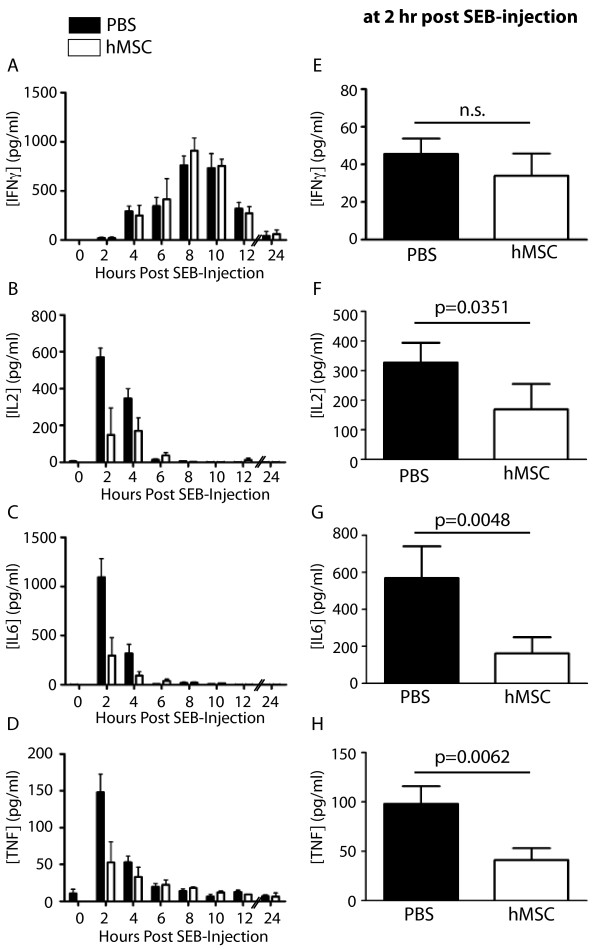
**hMSCs suppress proinflammatory cytokine production induced by SEB in vivo. (A-D)** Levels of IFNγ **(A)**, IL-2 **(B)**, IL-6 **(C)** and TNF **(D)** were measured from serum collected at the indicated times after SEB was injected in mice pre-treated with hMSCs (white) or PBS as a control (black). Each bar represents a mean + SEM. Statistical differences between the PBS and hMSC groups were assessed by 2-way ANOVA (p=0.6322 (A), p=0.0014 (B), p=0.0011 (C), p=0.0149 **(D)**, n=5/group/time). Data are representative of two independent experiments. **(E-H)** Serum levels of IFNγ **(E)**, IL-2 **(F)** and IL-6 **(G)** and TNF **(H)** at 2hr post-SEB injection were compared between PBS and hMSC groups. Statistical differences were assessed by the Mann–Whitney test. Data represent a pooled result of two independent experiments. n.s.=not significant.

In order to ensure their pluripotent potential, P3 hMSCs were differentiated and we show that these cells differentiated into the three predominant mesenchymal lineages: adipocytes, osteocytes and chondrocytes (Figure 1). MSCs have been shown to suppress production of pro-inflammatory cytokines and chemokines, including MIP-2(CXCL-2), CCL5, TNF, IL-6, and IL-1β, in animal models of lung injury and sepsis [16,18,22,27]. Therefore, we investigated whether hMSCs could modulate production of pro-inflammatory cytokines, including IL-2, IL-6, TNF and IFNγ previously implicated in the pathogenesis of experimental models of TSS [6,7,28-31]. Serum was collected from mice treated with hMSCs, or PBS as a control, at 2, 4, 6, 8, 10, 12 and 24 hours after peritoneal injection of SEB, and the serum levels of IL-2, IL-6, TNF and IFNγ were measured. Serum levels of all four cytokines rapidly increased following SEB injection, with the induction of IL-2, IL-6 and TNF preceding that of IFNγ (Figure 2A-D). Importantly, the levels of IL-2, IL-6 and TNF were significantly different between the PBS- and hMSC-treated groups (2-way ANOVA: p=0.0014(B), p=0.0011(C), p=0.0149(D)). No difference was observed between the groups in the level of IFNγ (Figure 2A, p=0.6332). When the two treatment groups were compared at different times, the serum levels of IL-2, IL-6 and TNF were significantly decreased in the hMSC-treated mice compared to the PBS-treated mice at 2 hours post SEB-injection (Figure 2E-H, p=0.0351(F), p=0.0048(G), p=0.0062(H)).

### Administration of hMSCs fails to improve survival in an experimental model of fatal staphylococcal TSS

**Figure 3:**
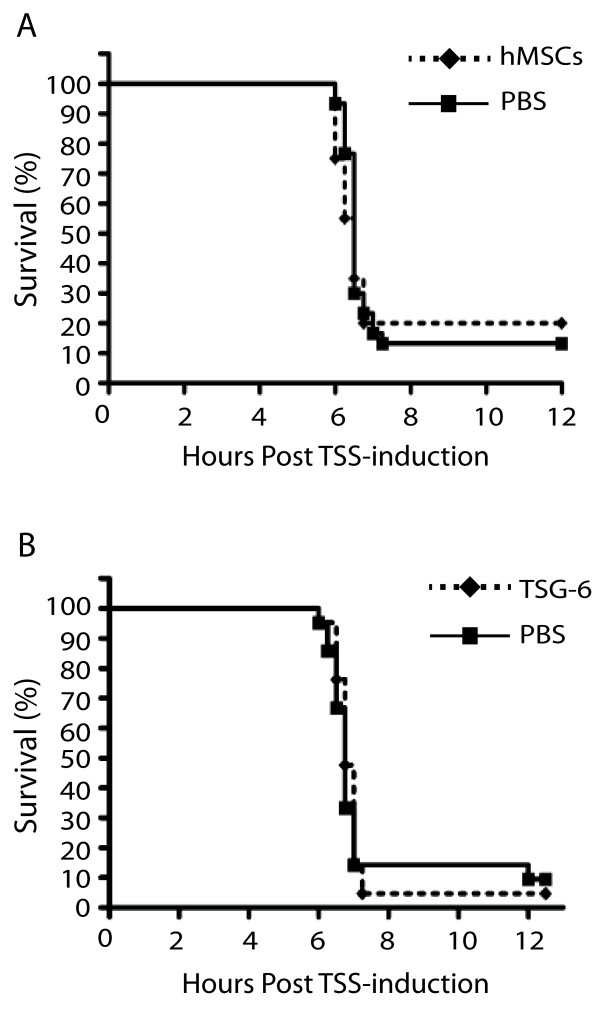
**Neither hMSCs nor TSG-6 improve survival in experimental fatal staphylococcal TSS.** Kaplan Meyer survival curves are shown for HLA-DR4 mice which were treated with 2.5×10^5^ hMSCs (**A**, dotted line, diamond), human recombinant TSG-6 (**B**, dotted line, diamond) or PBS (**A**, **B** solid, square) one hour prior to the induction of TSS. Statistical differences were assessed by log-rank test (p=0.7327 **(A)**, p=0.7258 **(B)**, n=20-22/group). Data represent a pooled result from two independent experiments.

Based on the finding that hMSCs suppressed serum levels of pro-inflammatory cytokines previously implicated in the pathogenesis of TSS, we hypothesized that MSCs would improve survival in experimental fatal TSS. To test this hypothesis, we used transgenic mice that express a chimeric MHC class II molecule consisting of HLA-DR-IEα and HLA-DRβ1*0401-1Eβ, which have a much higher affinity for superantigens than mouse MHC Class II molecules [23]. Therefore, unlike wild-type mice, the HLA-DR4 transgenic mice were demonstrated to develop TSS-like symptoms upon injection of a small amount of SEB (10 μg), following a sensitizing agent, D-galactosamine (D-gal) [7,24]. Chau et al. demonstrated that 100% lethality can be achieved after a single dose of 10 μg of SEB, following the sensitization with 30 mg of D-gal [24]. As D-gal is not involved in clinical TSS, the minimum amount of D-gal to achieve lethality was chosen for our study. The reduced dose of D-gal (10 mg) resulted in 80-90% lethality within 12 hours after the D-gal injection with 10 μg of SEB. Either SEB or D-gal at the indicated doses was not sufficient to induce lethality on its own (data not shown). Treatment of mice with hMSCs one hour prior to the induction of TSS did not significantly improve survival in this model (Figure 3).

MSCs can either stimulate or suppress host immune response depending on the host cytokine environment [19,32]. Therefore, we hypothesized that the administration of a known immune mediator produced by hMSCs would enable us to directly assess the immune-modulatory effects of this therapy independent of the host cytokine environment. TNF-stimulated gene 6 (TSG-6) is an anti-inflammatory glycoprotein that was shown to mediate the therapeutic effects of hMSCs in the animal models of myocardial infarction and zymosan-induced mouse peritonitis [15,33]. Therefore, we tested whether i.v. injections of human recombinant TSG-6 could improve survival in our TSS model. Human and mouse TSG-6 proteins share 92% sequence identity, and human recombinant TSG-6 has been shown to inhibit TNF expression in mouse macrophages in a co-culture experiment, and reduce inflammatory response and infarct size in a mouse model of myocardial infarction [15].

Both PBS-treated and TSG-6-treated groups showed 80-90% lethality with the median survival of 6.76 hours, and there was no statistically significant difference between these groups (Figure 3B).

### Administration of mMSCs also fails to improve survival in an experimental model of fatal staphylococcal TSS

**Figure 4:**
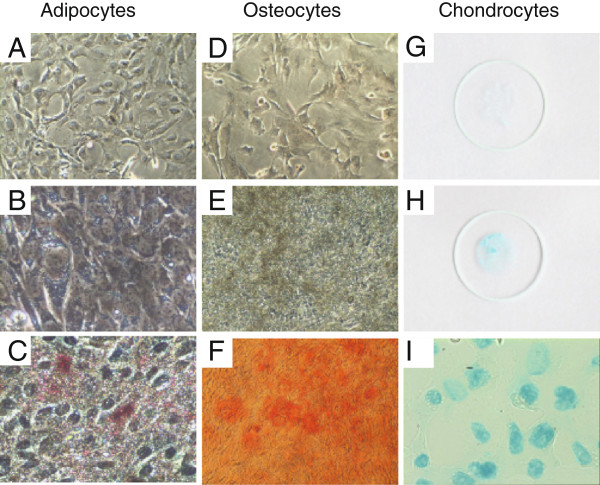
**Differentiation of Passage 6 mMSCs into adipocytes, osteocytes and chondrocytes.** Phase-contrast microscopy images of: adipogenic differentiation (**A**-**C**; Oil Red O staining of undifferentiated mMSCs **(A)** and differentiated adipocytes **(C)**; unstained differentiated adipocytes **(B)**); osteogenic differentiation (**D**-**F**; Alizarin Red S staining of undifferentiated mMSCs **(D)** and differentiated osteocytes **(F)**; unstained differentiated osteocytes **(E)**). **(G-I)** Alcian Blue staining of cytospins prepared from undifferentiated mMSCs **(G)** and differentiated chondrocytes **(H)**; phase-contrast microscopy image of the Alcian Blue stained cytospin of differentiated chondrocytes **(I)**.

**Figure 5:**
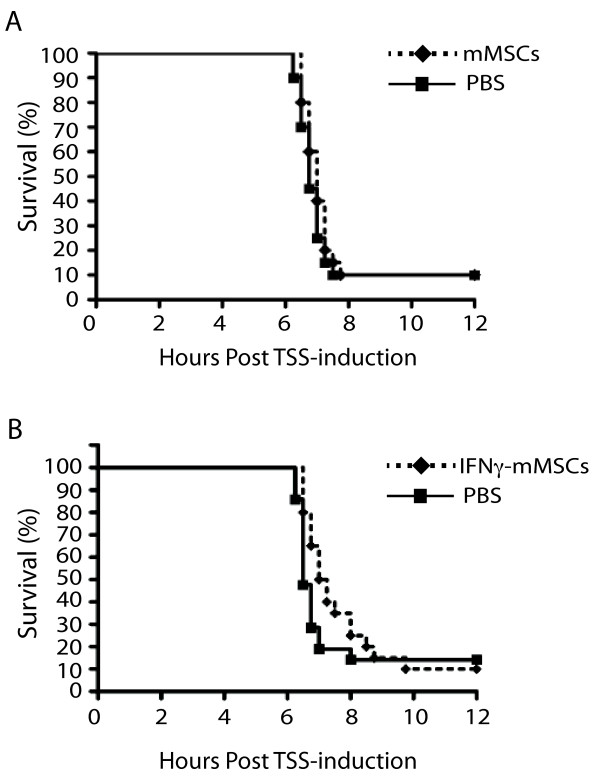
**mMSCs fail to improve survival in experimental fatal staphylococcal TSS with or without pre-treatment with IFNγ.** Kaplan Meyer survival curves are shown for HLA-DR4 mice which were treated with 2.5×10^5^ untreated mMSCs (**A**, dotted line, diamond), IFNγ-treated mMSCs (**B**, dotted line, diamond) or PBS (**A**, **B** solid line, square) one hour prior the induction of TSS. Statistical differences were assessed by log-rank test (p=0.4280 **(A)**, p=0.2184 **(B)**, n=20-22/group). Data represent a pooled result from two independent experiments.

In order to ensure that the lack of therapeutic efficacy of MSCs was not attributable to the heterologous use of human MSCs in a murine model we also examined whether mMSCs injected one hour prior to the induction of TSS could improve survival. The pluripotent potential of P6 mMSCs was confirmed by differentiating them into adipocytes, osteocytes and chondrocytes (Figure 4). However, similar to hMSCs we observed no significant survival benefit between the PBS- and mMSC-treated groups (Figure 5A). While the exact mechanism determining the immune suppressive function of MSCs is incompletely understood, multiple studies have underscored an important role for IFNγ in mediating these effects [21,32,34]. A previous study has demonstrated that the induction of IFNγ occurs relatively late in the experimental model of TSS induced by D-gal and SEB, and that the induction of IFNγ is preceded by the induction of TNF and IL-2, which is consistent with our observations (Figure 2) [6]. Therefore, we also assessed the effects of later administration of mMSCs on survival, i.e. when injected 3–4 hours after the D-gal injection. However, delayed mMSC treatment also failed to induce a survival benefit (data not shown).

Given the importance of IFNγ in eliciting an immunosuppressive phenotype of MSCs, we also investigated whether pre-treatment of mMSCs *ex vivo* with IFNγ prior to their injection into mice could improve survival. A previous study demonstrated that 100 U/ml of IFNγ represents a threshold above which mMSCs switch from an antigen-presenting phenotype to an immunosuppressive phenotype [32]. Therefore, we treated mMSCs with 100 U/mL of mouse IFNγ for 3 hours before harvesting them for injections. While there was a trend of increased median survival, this difference failed to reach statistical significance (Figure 5B).

## Discussion

This study used an experimental model to investigate the therapeutic potential of MSCs for TSS. We show that hMSCs suppressed circulating serum levels of IL-2, IL-6 and TNF induced by SEB, but not that of IFNγ. However, the immunomodulatory effect of hMSCs was insufficient to confer a survival benefit in a murine model of fatal TSS. Consistent with this finding, a downstream mediator produced by hMSCs, TSG-6, also failed to improve survival in experimental TSS. mMSCs were similarly incapable of improving survival even when pre-treated with IFNγ, which is thought to promote the immunosuppressive phenotype of MSCs [32]. Collectively, our data suggest that MSCs are unlikely to provide a therapeutic benefit against TSS despite their immune suppression of at least some of the implicated mediators of TSS.

Several novel treatment strategies for TSS have been proposed and are under investigation. Firstly, inhibiting the interaction of superantigens with the T cell receptor and MHC II has been explored by using peptide mimetics of superantigens [35,36], protein chimeras of the binding sites of superantigens [37], and nucleic acid aptamers which specifically bind superantigens [38]. Secondly, inhibitors have been developed against the key mediators of pro-inflammatory intracellular signaling pathways or pro-inflammatory cytokines and chemokines themselves [39-44]. Some of the experimental therapeutics have been previously approved by the FDA for other indications, receiving particular attention especially for biodefence purposes (e.g. rapamycin, dexamethasone, pentoxifylline) [42-45]. Finally, proof of concept of neutralizing superantigens by intravenous immunoglobulins (IVIg) has been demonstrated, and different recombinant antibodies are being investigated for their neutralizing potential although challenges remain with consistent neutralization of different superantigens [2,46-49].

The capacity of MSCs to suppress immune effector cells such as macrophages and T cells and their secreted mediators, as well as the relative ease of the isolation of MSCs from bone marrow, have brought much attention to the potential therapeutic application of MSCs to a range of human diseases. Among different models of diseases, therapeutic benefits of MSCs have mostly been reported in mouse models of acute lung injury and sepsis [16,18,22,27]. In these studies, administration of MSCs, either alone or with an antibiotic, improved survival and organ dysfunction, which was associated with reduced levels of pro-inflammatory cytokines (e.g., TNF, IL-6), chemokines, (e.g., CXCL2, CCL5 and KC/IL-8) in the peripheral blood and/or bronchoalveolar lavage fluid [16,18,22]. Our observation that hMSCs can suppress IL-2, IL-6 and TNF but not IFNγ is consistent with a previous finding in which administration of mMSCs reduced serum levels of IL-6 and TNF but not that of IFNγ in a cecal ligation and puncture model of sepsis [18].

Of note, the observed immunosuppressive effects on IL-2, IL-6 and TNF were not associated with an improved survival in experimental TSS in our study. There are several possible explanations for this observation. First, the difference in the host immune environment in the two models used for monitoring cytokine levels and for monitoring survival may result in a difference in the immunomodulatory properties of MSCs. MSCs are known to be highly sensitive to the host immune environment that can promote either an antigen presentation phenotype or alternatively, an immunosuppressive phenotype [19]. In our study, cytokine measurements were determined in wild-type C57BL/6 mice, in which only SEB was used to trigger TSS-like host immune response whereas the survival outcome was monitored in a lethal model of TSS, which required a sensitizing agent, D-gal, in addition to SEB.

It is possible that in the lethal model of TSS, the host immune environment is not optimal for inducing the immunosuppressive properties of hMSCs as in the non-lethal wild-type model. Alternatively, the lack of improvement in survival despite the partial immunosuppression by hMSCs may suggest that the hMSC-mediated immunosuppression does not affect the critical pathways and mediators for TSS-associated lethality. This latter hypothesis is supported by a recent report in which a neutralizing antibody against IFNγ significantly improved survival in SEB-induced TSS in HLA-DR3 mice, and the improved survival was associated with a reduction in the serum levels of chemokines, RANTES (CCL5) and KC (mouse CXCL1) [34]. Furthermore, in several earlier studies, therapeutic strategies that improved survival against experimental TSS were associated with suppression of IFNγ [41-43]. In our study, the suppressive effects of hMSCs were limited to IL-2, IL-6 and TNF, with no effect on IFNγ. The lack of effect of MSCs on IFNγ has also been observed by Nemeth et al. in a study where mMSCs significantly improved survival against experimental sepsis by reducing serum levels of TNF, and IL-6, but not IFNγ [18]. Previous findings by Tilahun et al. and others suggest that targeting IFNγ and its downstream mediators (e.g. CCL5, CXCL1) may be crucial in conferring a significant protection against experimental TSS [34,41-43].

There are limitations to our study. As an experimental model of human TSS, the HLA-DR4 transgenic mouse model requires D-gal as a sensitizing agent which induces an acute response that shares some but not all features of human TSS. A more recent model reported by Tilahun et al. uses HLA-DR3 transgenic mice in which lethality is induced without the need of a sensitizing agent [34], however, these mice are not commercially available. Directly correlating the immunomodulatory effects of MSCs with survival requires a time-course study examining cytokine levels in the lethal model used. However, there were technical constraints to sample appropriate numbers of animals given the rapidity of death in this model. Moreover, due to the requirement for a sensitizing agent in our model, we postulated that determining the effects of MSCs on SEB-mediated host immune response in the absence D-gal in the wild-type mice would be more relevant to understanding the immunomodulatory effects of MSCs on SEB-mediated TSS. Lastly, our study did not examine the effects of MSCs in experimental TSS induced by a Gram-positive bacterial strain, which may be more relevant to human TSS. Future studies assessing the effects of MSCs on TSS induced by Gram-positive bacterial strains in the HLA-DR3 model may provide additional insight into the immunomodulatory effects of MSCs on TSS.

The exact mechanism underlying the immunosuppressive effects of MSCs is poorly understood. Each infection state is characterized by distinct cytokine milieu that is dynamically regulated throughout the course of infection and is likely to influence whether MSCs can function as immunosuppressors in a given model. For instance, despite their capacity to suppress T cell proliferation *in vitro*, MSCs failed to improve clinical outcomes that are primarily mediated by T-cells *in vivo* in the models of heart transplant, graft versus host disease and collagen induced arthritis [50-52]. Similarly, hMSCs or mMSCs failed to improve survival in a murine model of severe influenza [53]. Indeed, in some cases, *in vivo* administration of MSCs was associated with increased levels of pro-inflammatory cytokines and poorer clinical outcome, depending on the dose and the time of administration [50,51]. In assessing therapeutic potential of MSCs, our present findings along with others, underscore the importance of elucidating the molecular targets of MSCs in a particular disease context, and their relevance to the pathogenesis of the disease.

## Conclusions

In summary, our data suggest that MSCs are unlikely to provide a therapeutic benefit for TSS. While MSCs can suppress some mediators of TSS, their immune suppressive capacity against TSS may be too limited quantitatively (i.e., duration and extent of suppression) and/or qualitatively (i.e. failure to suppress the critical mediators of TSS) to significantly alter the clinical outcome.

## Abbreviations

MSC: Mesenchymal stromal (stem) cells; TSS: Toxic shock syndrome; SEB: *Staphylococcal* enterotoxin B; MHC: Major histocompatibility complex; APC: Antigen presenting cells; D-gal: D-(+)-galactosamine hydrochlorde (D-gal); TNF: Tumor necrosis factor; IFN: Interferon; IL: Interleukin; TSG-6: TNF-stimulated gene-6.

## Competing interests

The authors declare that they have no competing interests.

## Authors’ contributions

HK designed, executed and analyzed *in vivo* and *in vitro* experiments and wrote the manuscript. ID and MM assisted with tail-vein injections. ID assisted with maintenance of the MSC culture. DJP provided MSCs and TSG-6. KK and WCL conceived the study, led the overall design of the study, and edited the manuscript. All authors have read and approved the final manuscript.
